# Multimodal nanoparticles as alignment and correlation markers in fluorescence/soft X-ray cryo-microscopy/tomography of nucleoplasmic reticulum and apoptosis in mammalian cells

**DOI:** 10.1016/j.ultramic.2014.05.009

**Published:** 2014-11

**Authors:** Christoph Hagen, Stephan Werner, Susana Carregal-Romero, Ashraf N. Malhas, Barbara G. Klupp, Peter Guttmann, Stefan Rehbein, Katja Henzler, Thomas C. Mettenleiter, David J. Vaux, Wolfgang J. Parak, Gerd Schneider, Kay Grünewald

**Affiliations:** a Oxford Particle Imaging Centre, Division of Structural Biology, Wellcome Trust Centre for Human Genetics, University of Oxford, Oxford OX3 7BN, UK; b Helmholtz Zentrum Berlin für Materialien und Energie GmbH, Wilhelm-Conrad-Röntgen Campus, 12489 Berlin, Germany; c Fachbereich Physik, Philipps Universität Marburg, Marburg 35043, Germany; d Sir William Dunn School of Pathology, University of Oxford, Oxford OX1 3RE, UK; e Institute of Molecular Virology and Cell Biology, Friedrich-Loeffler-Institut, 17493 Greifswald-Insel Riems, Germany

**Keywords:** cryoEM/T, electron cryo-microscopy/tomography, cryoFM, fluorescence cryo-microscopy, cryoXM/T, soft X-ray cryo-microscopy/tomography, eGFP, enhanced green fluorescent protein, N.A., numerical aperture, NEC, nuclear egress complex, NR, nucleoplasmic reticulum, PAH, poly(allylamine hydrochloride), PSS, poly(styrene sulfonate), Gold nanoparticles, Herpesvirus egress, Live-cell imaging, Nuclear membrane invaginations, Quantum dots, X-ray imaging

## Abstract

Correlative fluorescence and soft X-ray cryo-microscopy/tomography on flat sample holders is perfectly suited to study the uncompromised physiological status of adherent cells at its best possible preservation by imaging after fast cryo-immobilization. To understand the mechanism by which herpesviruses induce nucleoplasmic reticulum, *i.e.* invaginations of the nuclear envelope, during their egress from the host cell nucleus, morphologically similar structures found in laminopathies and after chemical induction were investigated as a potentially more easily accessible model system. For example, anti-retroviral protease inhibitors like Saquinavir also induce invaginations of the nuclear membranes. With the help of newly designed multimodal nanoparticles as alignment and correlation markers, and by optimizing fluorescence cryo-microscopy data acquisition, an elaborate three-dimensional network of nucleoplasmic reticulum was demonstrated in nuclei of Saquinavir-treated rabbit kidney cells expressing a fluorescently labeled inner nuclear membrane protein. In part of the protease inhibitor-treated samples, nuclei exhibited dramatic ultrastructural changes indicative of programmed cell death/apoptosis. This unexpected observation highlights another unique feature of soft X-ray microscopy, *i.e*. high absorption contrast information not relying on labeled cellular components, at a 3D resolution of approximately 40 nm (half-pitch) and through a sample thickness of several micrometers. These properties make it a valuable part of the cell biology imaging toolbox to visualize the cellular ultrastructure in its completeness.

## Highlights

• Nucleoplasmic reticulum was demonstrated in nuclei of Saquinavir-treated cells.
• New polyelectrolyte-Qdot^®^ 605 coated gold beads were employed as fiducials.
• Saquinavir can induce a strong apoptotic phenotype in the nucleus.
• CryoXT is an auspicious imaging technique in apoptosis research.

## 1 Introduction

Soft X-ray cryo-microscopy/tomography (cryoXM/T) is becoming an important and gap-bridging tool in the “*post-reductionist era of biochemistry*” [1], by directly imaging biological structures embedded in their native cellular environment to a resolution of a few tens of nanometers (recently reviewed, from a more technical point of view, for instance, in [2], and by [3]). Though hitherto mainly accessible at synchrotron facilities, new soft X-ray sources with a brightness close to early synchrotrons enable three-dimensional X-ray microscopy in the home laboratory [4], [5]. Having started later and, by that, lagging behind the technological development of electron cryo-microscopy/tomography (cryoEM/T) by decades, basic design of the light path and of the components of cryoXM apparatuses is still under extensive consideration and ongoing improvement [6], [7], [8]. Concerning the tomographic sample stage design of cryoXT end stations, different opinions have been presented. For example, Parkinson et al. [9] claimed that use of grids and other mounting systems developed for electron microscopy hinders full rotation and, therefore, a mounting system such as thin-walled glass capillary tubes is preferable. Otherwise, “*artifacts can be seen in the reconstruction due to missing data*” (quoted from [10]). Evidently, this “missing wedge” problem, which is well-known and inherent in flat specimen stage designs, did not hinder cryoET to produce groundbreaking results in structural biology (for a recent review, see, for instance, [11]). Alleviating techniques originally developed for electron (cryo-)tomography of flat samples, like dual axis or Saxton scheme tilt series acquisition [12], [13], [14], [15], might also help in cryoXT. Furthermore, taking shading/superposition in real samples into account, also the single-axis tomographic approach using a capillary sample holder misses data and does not result in isotropic resolution. Thus, elongated features oriented nearly perpendicular to the tilt axis suffer from a loss of resolution [16].

We consider different sample stage designs in cryoXT as practical alternatives, adequately chosen to match the biological object studied. Capillaries are perfectly suited for smaller particles/cells in suspension, like large viruses, bacteria, unicellular green algae, yeast or blood cells [17], [18], [19]. A certain and unavoidable reduction of the signal to noise ratio by the material of the capillary is balanced by full rotational data sampling, allowing for improved axial resolution in three-dimensional reconstructions. Flat, adherent cells such as mammalian cell monolayer cultures are better imaged on flat sample carriers/grids, serving as growth substrate. This way, detachment steps like trypsinization, necessary for loading them into capillary holders [9], can be omitted, thus leaving especially delicate and fragile cell lines like neurons intact. Otherwise, this harsh treatment might activate apoptotic pathways (examples showing dendrites/axons/synapses in cryoET, for instance, in Ref. [20], [21], [22]). Cells on supporting grids can readily be exposed to biological or chemical challenges, and incubation can be stopped at certain time points by cryo-immobilization without compromising the physiological status by further sample preparation. For example, PTK cells infected on-grid with vaccinia virus were analyzed by cryoXT, discriminating different maturation stages of the virus [23]. Here, we present results after incubation of mammalian cells on flat cryoXT grids with a therapeutic drug. Ultrastructural changes in the thickest cellular compartment, the nucleus, which is accessible to higher resolution imaging techniques like cryoET only after elaborate sample thinning procedures [24], [25], are characterized *in toto*.

Focusing on cell biological applications [26], the methodological development gained momentum by correlating cryoXM with fluorescence cryo-microscopy (cryoFM; for review, see Refs. [3], [27]). The power of this combined approach has recently been shown by localizing a fluorescently labeled protein within nanometer-resolved sub-cellular/sub-organelle structures. In that study, pUL34, the nuclear membrane-anchored component of the nuclear egress complex (NEC) of the *Herpesviridae* which interacts with viral pUL31, was found entering the perinuclear space in pUL34/pUL31 co-expressing mammalian cells by expanding the nucleoplasmic reticulum (NR) with vesicular structures induced by the NEC [28], [29]. The follow-up study presented here was designed to analyze functional and structural aspects of the nuclear envelope modifications occurring during herpesvirus nuclear egress (for a recent review, see [30]), by employing a biochemically well characterized and more easily accessible experimental model. Thus, human immunodeficiency virus protease inhibitors like Saquinavir that are part of HAART (highly active antiretroviral therapy) have been reported to also induce invaginations of the nuclear membranes [31]. These invaginations, so-called type I/II NR (for review, see [32]), are also known from laminopathies like the ageing disorder Hutchinson-Gilford progeria syndrome [33]. In parallel, we tested different multimodal nanoparticle designs as alignment and targeting/correlation markers for cryoXT (for recent review and applications not only in nano-imaging, see [34], [35]). Although only partly serving the biological purpose of this study, *i.e.* to provide a robust experimental model for induction and correlated cryoFM/cryoXT characterization of type I/II NR, our results from Saquinavir treated cells give new insights into programmed cell death/apoptosis, a cellular process not yet studied by cryoXM/T.

## 2 Material and methods

### 2.1 Cells and incubation

Rabbit kidney (RK13) cells expressing the N-terminal 285 amino acids comprising the nucleoplasmic tail and the first transmembrane span of human lamin B receptor protein, fused to eGFP (enhanced green fluorescent protein), were generated by transfection with plasmid pLBR1TM-GFP [36] by calcium phosphate co-precipitation [37] and selection with 0.5 mg/ml G418. Stable eGFP-positive cell clones showing nuclear rim staining were isolated by aspiration and further characterized. For the incubation experiments described here, this cell line (catalog no. RIE 1213 of the Collection of Cell Lines in Veterinary at the FLI, Greifswald-Insel Riems, Germany) was grown in Dulbecco׳s modified Eagle medium (Gibco-Invitrogen, Karlsruhe, Germany) supplemented with 10% (w/v) fetal calf serum and 1% (v/v) PSN Antibiotic Mixture (Gibco-Invitrogen). HeLa cells (ATCC CCL-2, human cervical adenocarcinoma cells) transiently expressing eGFP-tagged lamin B1 were cultivated as described above, and details for their transient transfection protocol are given in Ref. [38].

Saquinavir (mesylate) was provided by the NHS Reagent Program (https://www.aidsreagent.org) and was prepared as a 5 mM stock, either in methanol or in dimethyl sulfoxide (DMSO). We found the latter stock solution yielding a stronger reaction during incubation. That might be related to a lower solubility of Saquinavir in methanol as compared to DMSO [39]. Controls were incubated with the corresponding concentration of the solvent only. All incubation steps were performed directly with the cells growing on the perforated carbon foil of the HZB-2 gold grids arranged in plastic microscope slide growth chambers (µ-slide 2×9 well, Ibidi GmbH, Munich, Germany; [29]).

### 2.2 Preparation of the nanoparticles

Size-tunable photoluminescent aqueous CdSe/ZnS (emission maximum: 625 nm) microspheres were prepared as described [40].

Multilayer polyelectrolyte-Qdot^®^ 605 coated (commercial quantum dots with emission maximum at 605 nm; Invitrogen # Q21701MP) gold beads were prepared essentially as described [41]. Firstly, commercial gold nanoparticles with mean core diameter 198±13 nm (as measured from transmission electron microscopy images), a hydrodynamic diameter of 210±2 nm and a ζ-potential of −20.3±0.7 mV (both measured in Milli-Q water with dynamic light scattering in a zetasizer) were coated with several layers of polyelectrolytes with opposite charge by means of the Layer-by-Layer (LbL) approach [42]. Poly(styrene sulfonate) (PSS) and poly(allylamine hydrochloride) (PAH) were used as the negative and the positive polyelectrolyte, respectively. Nine layers of polyelectrolytes were added on the gold beads (*i.e*. Au@(PAH/PSS)_4_ PAH) making their surface positively charged. It is known that the presence of metallic nanostructures in close proximity to quantum dots can modify their photoluminescence properties [43], [44]. The number of polyelectrolyte layers was chosen to avoid the quenching of the Qdot^®^ 605 nanoparticles since every layer has been reported to have a thickness between 0.5 and 2 nm [45], [46]. Qdot^®^ 605 nanoparticles were functionalized with the polymer poly(isobutylene-alt-maleic anhydride) as reported elsewhere [47] and therefore they were negatively charged (the ζ-potential and hydrodynamic diameter measured in Milli-Q water were −35.3±0.6 mV and 19±2 nm, respectively). Negatively charged Qdot^®^ 605 nanoparticles were attached on the gold beads due to electrostatic interactions. One last layer of PSS was added to the core-shell structure. The final hydrodynamic diameter and ζ-potential were 277±4 nm and −9.5±0.9 mV, respectively. Nanoparticle suspensions were concentrated by gentle centrifugation or by sedimentation overnight. A detailed “*Protocol for the synthesis and characterization of polyelectrolyte-Qdot*^®^ *605 coated gold beads*” is provided in the Supporting information.

### 2.3 Sample preparation for fluorescence and soft X-ray cryo-microscopy

A detailed “*Protocol for partially coherent X-ray microscopy*” is provided in the Supplement of [48]. Improvements or alternative procedures of the sample preparation steps for cryoXM/T at the HZB TXM at beamline U41-FSGM of the BESSY II electron storage ring in Berlin/Germany, including live-cell microscopy and cryo-immobilization by plunge freezing, are explained in Ref. [29], and were employed here. Before cell seeding and incubation, one microliter of the respective highly concentrated nanoparticle suspension was applied to the carbon-coated mesh area of the grids, and, additionally, two microliter of a 1:4 dilution in culture medium on top of the grown/incubated cells, immediately before blotting and plunge freezing.

### 2.4 External and in-column light cryo-microscopy

External cryoFM was performed with a second-generation cryo-holder (Cryostage^2^) essentially as described in [49]. In order to avoid getting objectives in touch with the rim of the dedicated objective cavity underneath the Cryostage^2^ during changes of magnification, the working position of the motorized stage (DC 120×100, Märzhäuser, Wetzlar, Germany) on an AxioObserver.Z1 inverted microscope (equipped with HXP 120 for fluorescence excitation and an AxioCam MRm for detection; Carl Zeiss MicroImaging GmbH, Göttingen, Germany) was statically brought close to its upper focus limit. After transferring the vitreous sample on a HZB-2 grid into the modified sample holder of the Cryostage^2^ (pre-cooled with a Norhof LN_2_ Microdosing system, Series 900, Maarssen, The Netherlands), an automated three channel grid scan was performed (phase contrast; eGFP: eGFP HQ filter set F36-528 from AHF analysentechnik AG, Tübingen, Germany; nanoparticles: Texas Red ET filter set F46-008 from AHF), first with a 20× objective (numerical aperture, N.A. 0.4), and the latter two detection channels then separately, due to exceeding file size, with a long working distance 63× objective (N.A. 0.75).

A technical outline of the in-column epi-fluorescence and reflected light microscope of the HZB TXM is provided by Schneider et al. [3], and was deployed for this study as detailed in Ref. [29].

### 2.5 Soft X-ray cryo-microscopy/tomography at the HZB TXM at beamline U41-FSGM of the BESSY II electron storage ring in Berlin/Germany

Details of data acquisition and analysis are given in [29]. Targeting on areas in the vitreous sample suitable for tomography was guided by external or, additionally, in-column cryoFM data. Soft X-ray exposure was set to 4 s or longer to average illumination fluctuations as much as possible, and the monochromator slit was adjusted to exploit only one third of the full dynamic range of the camera, in order to avoid radiation damage during tilt series acquisition. Tilt series presented here were taken with the 25 nm zone plate objective (object pixel size: 9.9 nm), from −60° to 60° tilt angle with 1° spacing, by default.

Tomographic reconstruction was performed using the Etomo GUI of IMOD [50], [51]. If not stated otherwise, tomograms and slices presented here are binned with a kernel of 2×2×2, and slices are shown in the corresponding voxel size thickness. Visualization was performed with Amira 5.2 (Visage Imaging GmbH, Berlin) and Adobe Photoshop CS4 (Adobe Systems Inc.).

## 3 Results

### 3.1 Photoluminescent aqueous CdSe/ZnS microspheres as fiducial markers

**Fig. 1:**
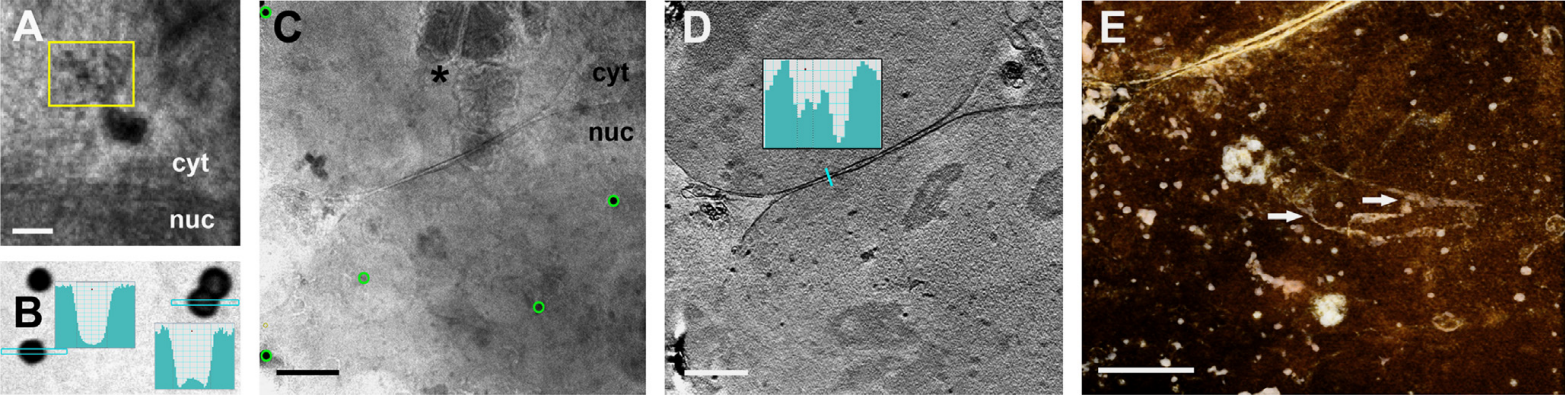
Quantum dot-containing nanoparticles as alignment markers in cryoXT of vitreous mammalian cells. (A) Sub-area of the zero degree image taken from a tilt series of a nucleus of a rabbit kidney epithelial-like RK13 cell (sample thickness: 8 µm) stably expressing LBR1TM-GFP (*cf.* Supplement Movie 1, pre-aligned tilt series). Note the low absorbance contrast of photoluminescent aqueous CdSe/ZnS microspheres (*λ*_*max.em*._=625 nm; diameter: ~150 nm; yellow frame) as compared to standard gold-coated silica beads (center). (B) Density profiles of the other tested multimodal nanoparticles, polyelectrolyte-Qdot^®^ 605 coated gold beads (left; diameter: 208 nm) and standard gold-coated silica beads (right; diameter: 267 nm) in a zero degree image of a cryoXT tilt series (sample thickness: 3 µm). Note that the polyelectrolyte-Qdot^®^ 605 coat *(λ*_*max.em.*_=605 nm) of the former nanoparticles, although imaged without cellular background in a hole of the Quantifoil carbon coat of the grid, did not provide sufficient contrast to be detected separately from the gold core (total diameter of the nanoparticles measured by dynamic light scattering: 277 nm, standard deviation 8 nm; *cf*. Supporting information). (C–E) Soft X-ray cryo-tomography of two nuclei before cytokinesis in a RK13 cell expressing LBR1TM-GFP, incubated on-grid with 20 µM Saquinavir for 48 h. The tilt series was aligned with the help of polyelectrolyte-Qdot^®^ 605 coated gold beads as alignment markers (green circles in C: three polyelectrolyte-Qdot^®^ 605 coated gold beads in the periphery of the image frame, and two cellular marker points, most probably lipid bodies, were used for alignment; asterisk: ice contamination on the sample surfaces; *cf*. Supplement Movie 2, pre-aligned tilt series). The next panels show the corresponding reconstruction (D, cyan bar: measuring position of the density profile shown as inset; profile determined from unbinned data, pixel size: 9.9 nm, revealing the inner and outer nuclear membrane of the upper nucleus with a distance of 40 nm; *cf*. Supplement Movie 3, sliced view through the tomographic reconstruction; thickness of reconstructed volume: 4.1 µm) and visualization as rendered volume with inverse contrast (E, arrows: nucleoplasmic reticulum; Supplement Movie 4, animated sub-volume of the lower nucleus). Scale bars are 500 nm (A) and 2 µm (C–E). (For interpretation of the references to color in this figure legend, the reader is referred to the web version of this article.)

Fiducial-marker based alignment of tomographic tilt series is the ‘gold’ standard to assure highest possible resolution in soft X-ray as well as in electron cryo-tomograms. Thus, finding chemically inert nanoparticles providing high soft X-ray contrast, with a diameter of approximately 200 nm and, preferably, fluorescence for correlative microscopic purposes, is of high priority (Fig. 1). Good candidates exhibiting a bright fluorescence signal which were thought, potentially, being more resistant to bleaching by soft X-ray radiation than the fluorophores in our earlier study [29], are quantum dots like in size-tunable photoluminescent aqueous CdSe/ZnS microspheres [40]. Such microspheres are composed of oligomers of amphiphilic polymaleic acid n-hexadecanol ester, with multiple embedded CdSe/ZnS quantum dots. They were tested as alignment markers in cryoXT of vitreous adherent cells (Fig. 1A). In their current shape, *i.e*. ~150 nm in diameter in which the CdSe/ZnS quantum dots are not adding much soft X-ray contrast to the polymeric spheres, the center of each microsphere could not be determined reliably throughout the full tilt series, thus preventing high-quality alignment of the images at least for thicker samples (>5 µm; *cf*. Supplement Movie 1). However, reliable marker particle tracking was possible with standard gold markers (*cf*. [48]), consisting of silica-spheres with a gold shell, ~270 nm in diameter [52], up to high tilt angles where, due to limits in the depth of focus of the zone plate objective of the soft X-ray microscope, images get blurred in regions off the tilt axis (Fig. 1A, Supplement Movie 1). Still, the fluorescence of the aqueous CdSe/ZnS microspheres was very bright (peak emission wavelength: 625 nm; [40]), possibly allowing to localize single nanoparticles in correlative cryoFM. Thus, we consider it worthwhile to improve the performance of these nanoparticles, by enlarging the diameter to at least 200 nm, and by increasing the concentration of CdSe/ZnS quantum dots in the polymeric microspheres. Additionally, polystyrene microspheres with embedded CdSe/ZnS quantum dots were tested without success, due to low contrast and even smaller size (~80 nm in diameter; data not presented).

### 3.2 Polyelectrolyte-Qdot^®^ 605 coated gold beads as fiducial markers

Multilayer capsules with a massive gold core (Fig. 1B) and a polyelectrolyte shell comprising quantum dots performed similarly well, and sometimes even better, as alignment markers compared to the standard silica-spheres with a gold shell (*cf*. [48]; Fig. 1C–E). As exemplified along the data analysis steps of cryoXT in cells treated with Saquinavir, the high contrast and, thus, excellent traceability of the polyelectrolyte-Qdot^®^ 605 coated gold spheres in the tomographic tilt series (Fig. 1C; Supplement Movie 2) added to a low residual error mean of the alignment (0.78, standard deviation: 0.47). This resulted in a tomographic reconstruction revealing structural details at a 3D resolution close to the practical limit (*cf*. [29]), like visualization of the two membranes of the nuclear envelope (Fig. 1D, Supplement Movie 3), and the NR running through the nucleus, in a reconstructed subvolume (Fig. 1E, Supplement Movie 4).

**Fig. 2:**
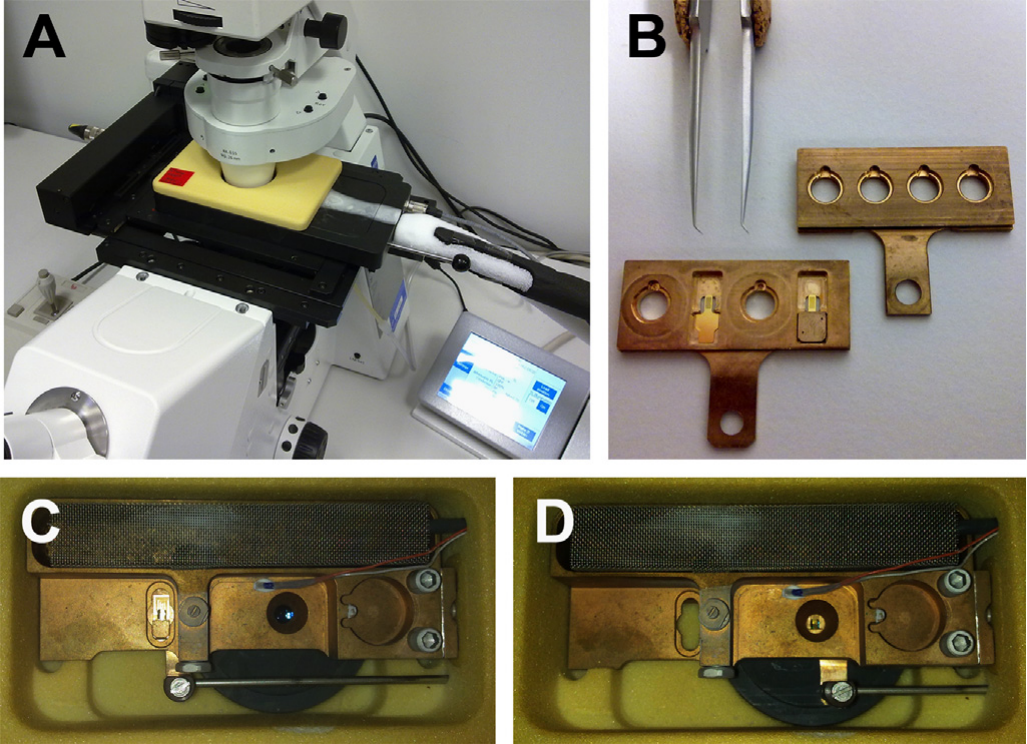
Modification of Cryostage^2^ (MPI Biochemistry, Martinsried) to enable correlative fluorescence cryo-measurements of vitreous samples, on dedicated grids (IFR-1 or HZB-2) for the soft X-ray cryo-microscope at the beamline U41-FSGM of BESSY II (Berlin). (A) Overview of the Cryostage^2^ mounted in a motorized microscope stage of an inverted light microscope. (B) Modified specimen slider of the Cryostage^2^ (left, with HZB-2 gold grids in position 2 and 4) in comparison to the original slider for 3.05 mm standard electron microscopic grids only (right). (C) Loading and (D) imaging position of a HZB-2 gold grid in the sample chamber of the Cryostage^2^.

**Fig. 3:**
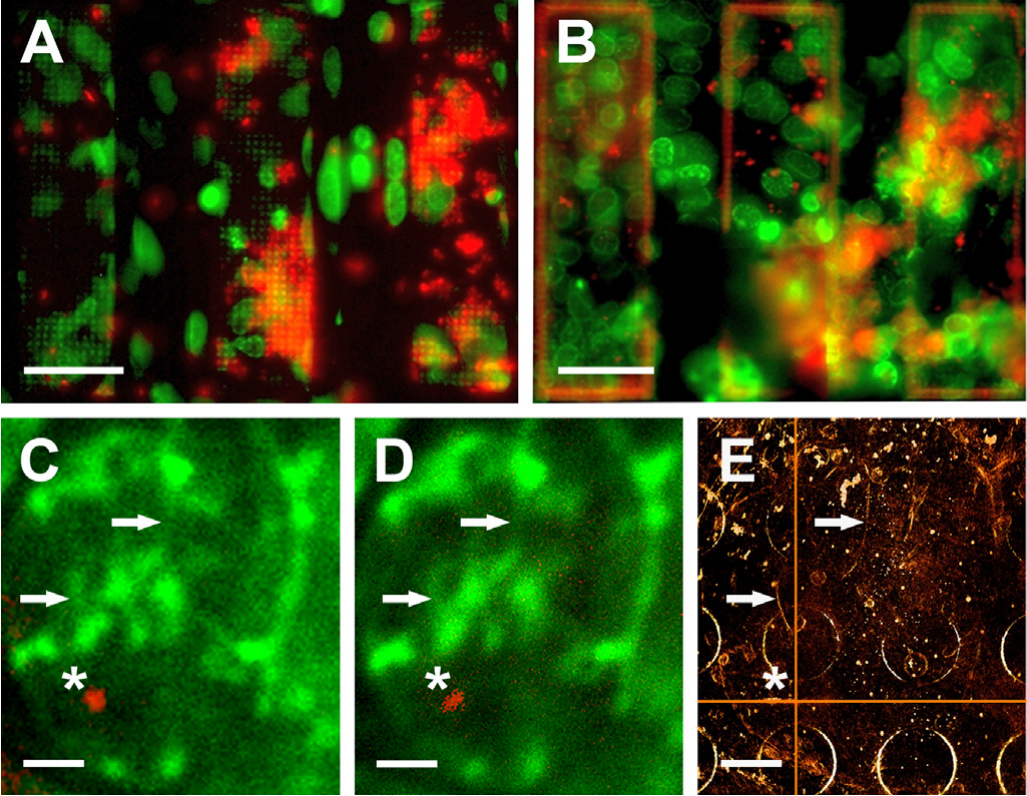
Polyelectrolyte-Qdot^®^ 605 coated gold beads as correlation markers in fluorescence and cryoXT. RK13 cells stably expressing the (inner) nuclear membrane marker protein LBR1TM-GFP (green channel in A–D) were incubated on a Quantifoil R2/2 carbon-coated HZB-2 gold grid with 20 µM Saquinavir for 48 h. Polyelectrolyte-Qdot^®^ 605 coated gold beads (*λ*_*max.em.*_=605 nm; red channel in A-D) were applied to the carbon support film before cell seeding and incubation. Pre-scanning of the sample was performed with a 20× objective (N.A. 0.4) at 37 °C in an inverted setup, *i.e.* the perforated carbon support foil facing the objective lens, 4 h before vitrification (A), and after vitrification at −170 °C on a modified Cryostage^2^ with the cells facing the objective lens (B; the same grid slots as in A are depicted). Note the shading/absorption of epi-fluorescence by the support foil in A. (C-E) Correlation of fluorescence and cryoXT data in a nucleus of a Saquinavir-treated RK13 cell. Fluorescence was acquired externally on a Cryostage^2^ (C; objective: 63×, N.A. 0.75), and in-column of the HZB TXM (D; objective: 100×, N.A. 0.75). The lateral position of a single polyelectrolyte-Qdot^®^ 605 coated gold bead is marked by an asterisk in C-E, and additionally by orange XY-lines in the (rendered) volume of the X-ray tomogram (C–E, arrows: nucleoplasmic reticulum; *cf.* Supplement Movie 5, dynamic superimposition of D and E; Supplement Movie 6, sliced view through the tomographic reconstruction; thickness: 2.8 µm). Scale bars are 50 µm (A and B) and 2 µm (C–E). (For interpretation of the references to color in this figure legend, the reader is referred to the web version of this article.)

In order to perform cryoFM for subsequent correlation with cryoXT of the vitreous specimens, and to save precious beam time at the HZB TXM in Berlin, the Cryostage^2^ (MPI of Biochemistry, Martinsried, [49]) was modified to support the use of HZB-2 grids (Fig. 2). Thus, plunge frozen samples can be pre-scanned and pre-selected in the home laboratory, increasing search efficiency at the HZB TXM, and in advance providing fluorescence data for later correlation, at a similar resolution as it can be achieved with the in-column light cryo-microscope of the HZB TXM in Berlin (Fig. 3; *cf*. [3], [29]). The red fluorescence of the polyelectrolyte-Qdot^®^ 605 coated gold beads was detectable under cell growth conditions (37 °C; Fig. 3A) and in vitreous samples at approximately −170 °C (Fig. 3B). The fluorescence signal was strong enough to localize single nanoparticles with our equipment for external or in-column correlative cryoFM (long working distance objectives; Fig. 3C–E). In the analyzed nucleus of a Saquinavir-treated RK13 cell stably expressing the (inner) nuclear membrane marker protein LBR1TM-GFP, a part of the green fluorescing structures was found to correlate with the tubular structures of the NR seen by soft X-ray data, demonstrating the membranous character of these tubes (Fig. 3C–E, Supplement Movie 5, Movie 6).

**Fig. 4:**
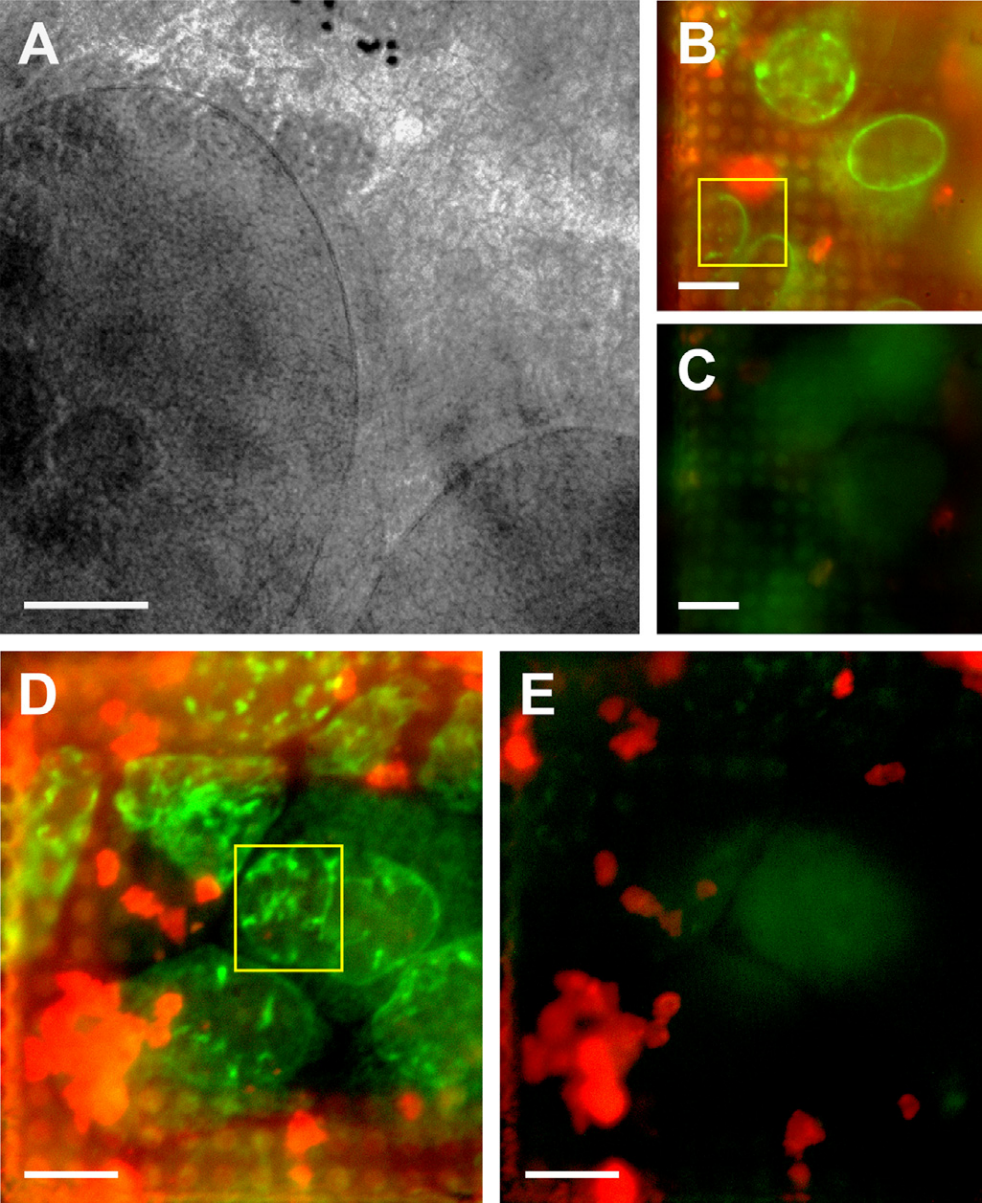
Fluorescence of polyelectrolyte-Qdot^®^ 605 coated gold beads gets bleached by soft X-ray irradiation during tomographic data acquisition (red channel in B–E). (A) Zero degree image of a tilt series. Note the bead aggregate, embedded in a grainy matrix, in the upper part of the image (*cf*. Supplement Movie 7, pre-aligned tilt series). (B–E) Fluorescence was acquired from two areas of a sample of RK13 cells stably expressing LBR1TM-GFP (green channel in B–E), incubated on a Quantifoil R2/2 carbon-coated HZB-2 gold grid with 20 µM Saquinavir for 48 h, in-column of the HZB TXM before (B and D) and subsequently to soft X-ray exposure of ~10^9^ Gy (C, E; *cf.* Supplement Movie 8, dynamic superimposition of B and C, and Supplement Movie 9, dynamic superimposition of D and E). As camera settings were kept exactly the same, it demonstrates that fluorescence of aggregated (B and C) and single polyelectrolyte-Qdot^®^ 605 coated gold beads (D and E), applied in excess as alignment and correlation markers before cell seeding and incubation, was bleached almost completely. The yellow square in B marks the camera frame area at 0° tilt angle of the tilt series depicted in A. The yellow frame in D marks the area of the tilt series shown in Fig. 3E and Supplement Movie 6. Scale bars are 2 µm (A) and 10 µm (B–E). (For interpretation of the references to color in this figure legend, the reader is referred to the web version of this article.)

Comparison between in-column cryoFM before and after cryoXT data acquisition showed that the fluorescence of quantum dots was strongly diminished by soft X-ray irradiation (Fig. 4A–E), resembling earlier observations for fluorescence of GFP and of FluoSpheres [29]. Occasionally, red fluorescing masses were found, and non-fluorescent nanoparticles were also observed. This might indicate a partial instability of the Qdot^®^ 605-containing polyelectrolyte shell. Additional purification procedures and/or more gentle concentration steps avoiding centrifugation might help to improve the marker preparation.

### 3.3 Apoptosis in Saquinavir-treated nuclei

**Fig. 5:**
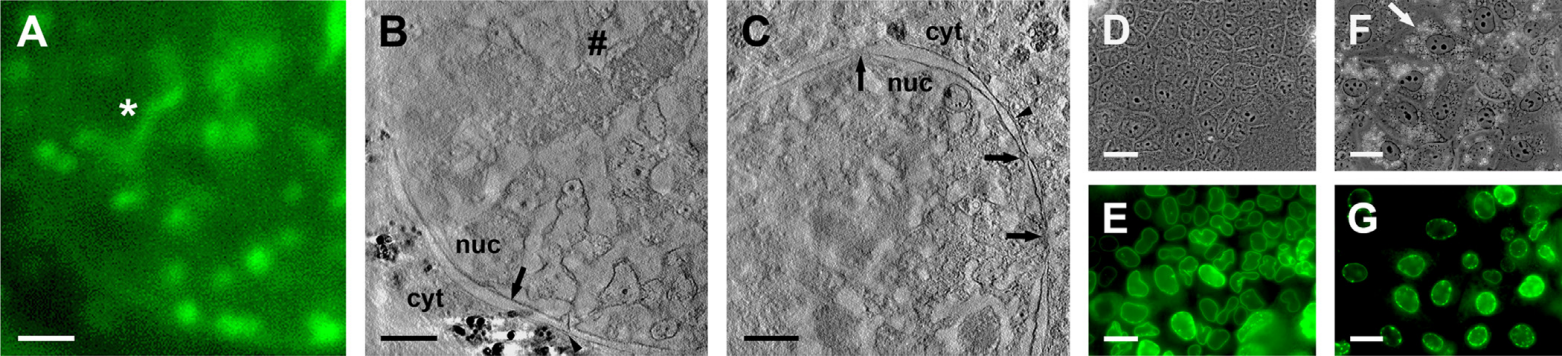
Apoptosis observed by cryoXT. In RK13 cells expressing LBR1TM-GFP (green channel in A, E and G) and incubated with 20 µM Saquinavir for 24 h, linear structures, such as for Fig. 3C–E, in in-column fluorescence cryo-microscopic images (A; asterisk) were not matched by tubular structures in the soft X-ray cryo-tomogram of the same nucleus (B; *cf*. Supplement Movie 10, sliced view through the tomographic reconstruction; total thickness: 2.6 µm), structures possibly marking the remnants of chromosome territories appeared (B, hashtag), and the chromatin area was clearly detached from the nuclear envelope (the nuclear envelope is marked by arrowheads in B and C; note outer and inner nuclear membrane, *cf.* Supplement Movie 10, Movie 11) and was confined by a boundary of unknown origin (B, arrow). Sometimes, the nuclear envelope was found open to the cytoplasm (C, arrows; *cf.* Supplement Movie 11, sliced view through this tomographic reconstruction; thickness: 1.8 µm). As compared to the control (D), live cell-microscopic phase contrast images revealed massive cytoplasmic vesiculation in RK13 cells exposed for 48 h to 20 µM Saquinavir (F, arrow). In the GFP-fluorescence channel of these live cell-microscopic measurements, ‘speckles’ in the nuclei of the treated samples were more prevalent (G), as compared to the control (E; *cf.* Supplement Movie 12, dynamic superimposition of D and E, and Supplement Movie 13, dynamic superimposition of F and G). Scale bars are 2 µm (A–C) and 20 µm (D–G). (For interpretation of the references to color in this figure legend, the reader is referred to the web version of this article.)

In this study, we applied Saquinavir, an antiviral therapeutic protease inhibitor known to also cause prelamin A accumulation as a side effect [31] and, therefore, to induce changes in the nucleus which are comparable in ultrastructure to what we have observed in cells co-expressing herpesvirus NEC proteins pUL31 and pUL34 [29]. Unexpectedly, in most of the cells treated with a moderate Saquinavir concentration, a clear apoptotic phenotype was observed with compacted and segregated chromatin masking other structural changes in the nucleus almost completely (*cf*. Table 1). Thus, fluorescent proteins anchored to the inner nuclear membrane (here: lamin B receptor construct, LBR1TM-GFP; [36]) were no longer usable as a reliable marker to find tubes of NR in correlated cryoXT data (Fig. 5A and B; Supplement Movie 10). In these apoptotic nuclei, we observed detachment of the chromatin area from the nuclear envelope with a distinct boundary of unknown origin but not involving the inner nuclear membrane, as shown by the still intact double membranes of the nuclear envelope observed regularly at least in the higher resolved parts of the tomograms (Fig. 5B, C, arrowheads; *cf.* Supplement Movie 10, Movie 11). Aside from partial disintegration of both nuclear membranes (Fig. 5C, Supplement Movie 11), another apparent change in the overall structure of the apoptotic nuclei was their exceptional flatness on the growth substrate (thinnest measurement: 1.5 µm). Typical symptoms for apoptotic/autophagic processes in Saquinavir-treated samples, such as extensive vesiculation in the cytoplasm, were also observed by live-cell microscopy in control experiments (Fig. 5D–G; Supplement Movie 12, Movie 13), and in soft X-ray cryo-tomograms of HeLa cells transiently expressing GFP-tagged lamin B1, after 48 h of incubation with 20 µM Saquinavir (Supplement Movie 14).

**Table 1:** Number of apoptotic/non-apoptotic nuclei observed in cryoXT data of vitreous RK13 cells expressing LBR1TM-GFP, in dependence on incubation with 20 µM Saquinavir.

	Time of incubation	
	24 h	48 h
Control	n.d.	2^a^/10^b^
20 µM Saquinavir, methanol stock	n.d.	4/5
20 µM Saquinavir, DMSO^c^ stock	15/0	n.d.

a Number of nuclei exhibiting apoptotic phenotype.

b Number of nuclei exhibiting no signs of apoptosis.

c Dimethyl sulfoxide.

## 4 Discussion

Our experience with methods for fiducial-less alignment of tomographic tilt series, based on the patch tracking mode of IMOD/Etomo GUI [50] and a MATLAB implementation of algorithms described in [53] in combination with the global alignment correction capabilities of IMOD [51], confirm that manual alignment of cryoXT tilt series using fiducial markers provides in most cases better results than automated feature tracking methods, especially in thicker samples [54], [55]. Thus, the development of efficient fiducial markers for cryoXT is important to achieve high resolution in tomographic reconstructions (Fig. 1). Furthermore, a combination of high soft X-ray contrast with fluorescence emission in the very same multifunctional nanoparticle facilitates correlative cryoFM/XT approaches (Fig. 3), potentially providing multimodal markers to enable a localization/correlation accuracy with high precision, as it has been demonstrated recently for cryoFM/EM [56], [57]. Thus, the added value of having both features, fluorescence emission and soft X-ray absorption, in one nanoparticle together is that cryoFM and cryoXM data can be spatially registered to a common coordinate system, allowing for high statistical confidence in correlating molecule-specific with structural (context) information. For this purpose, small point-like markers are better suited than the regular two-micrometer holes in the Quantifoil carbon growth support that can be seen in all detection modalities, too [29]. In our experiments, the expectations toward high soft X-ray contrast and high fluorescence emission efficiency of the markers were fulfilled by a design with a massive gold core and quantum dots embedded in a multilayered shell around the core. Optimal thickness of the latter and, thus, ensuring a certain distance between quantum dots and the surface of the gold spheres to prevent fluorescence quenching [58], was achieved by variation in the number of polyelectrolyte layers. An increase in mechanical and chemical stability of the multilayered nanoparticles, for instance in solutions with higher ionic strength like cell growth media, might be achieved by addition of a final silica layer.

Soft X-ray stability of quantum dots as fluorescent markers in biological cryoXM has been studied before [59], [60]. Since fluorophores like organic dyes or genetic marker/tags are functionally destroyed by rather low doses of soft X-ray irradiation, our results demonstrating strong soft X-ray-induced photobleaching also of quantum dot fluorescence reconfirm that acquisition of fluorescence data is required before any X-ray exposure ([29]; Fig. 4). In addition to the establishment of an in-column epi-fluorescence and reflected light cryo-microscope in the HZB TXM in Berlin [3], this is now supported by expanding the sample spectrum of the Cryostage^2^ to IFR-1 or HZB-2 grids in a more reproducible way than it has been shown using the former Cryostage model from the Baumeister lab in Martinsried ([61]; *cf*. Fig. 2). Thus, in preparation for analysis at the synchrotron beamline, target structures and sample spots suitable for cryoXT can be pre-selected in the home laboratory. This has also been demonstrated recently employing a non-motorized cryo-stage (Linkam Scientific Instruments, Epsom, UK; *cf*. [62]). High-numerical aperture cryo-objectives are not commercially available, and the described light/fluorescence cryo-microscopes are restricted to long- or cooled short-working distance air objectives (for technical overview, see [63]), currently achieving a N.A. of 0.95 [56]. The only application of a cryo-immersion objective described so far, claiming a final N.A. of 1.3 under cryo-conditions, is based on the combination of two objectives of manufacturers that went out of business already in the first half of the last century [64].

In this study, newly designed polyelectrolyte-Qdot^®^ 605 coated gold beads helped us as tomographic alignment and fluorescent correlation markers to visualize an elaborate three-dimensional network of the LBR1TM-GFP-labeled NR in nuclei of Saquinavir-treated rabbit kidney cells (Fig. 1, Fig. 3). The overall features of this network, comprising mostly flat and tubular invaginations of the nuclear envelope, were in accordance to fluorescence and conventional electron microscopy data presented before in comparable experimental systems [32], [33]. In our previous study in viral pUL34/pUL31 co-expressing mammalian cells, the NR tubes were filled with vesicular structures induced by the NEC [29]. Unfortunately, due to their strong apoptotic phenotype, nuclei of Saquinavir-treated cells were mostly not suitable to study the NR by correlative cryoFM/XT in detail. We interpret the dramatic structural changes in the nucleus, revealed here by cryoXT, as apoptosis because Saquinavir is known to induce this highly regulated and energy-dependent cellular process [65], and the characteristics observed by cryoXT were in accordance with general morphological changes in apoptotic nuclei (for review, see [66]; Fig. 5). Thus, the process of apoptotic nuclear dismantling includes events affecting the nuclear envelope, such as detachment from chromatin and proteolysis of nuclear membrane and pore proteins, as well as proteolysis of the nuclear matrix and chromatin condensation in coordination with DNA fragmentation [67]. However, the concentration of Saquinavir applied in our study (20 µM), yielding an apoptotic morphology in most of the nuclei of our cells after 24 h of incubation, was inefficient to induce apoptosis/autophagy in a panel of ovarian cancer cell lines [39]. This might be explained by the fact that stable overexpression of the LBR1TM-GFP tag, to observe the NR in fluorescence microscopy, made the RK13 cells more sensitive for induction of apoptosis (*cf*. Table 1, controls). Additionally, it might point to a higher sensitivity of cryoXM to detect apoptotic changes in the nucleus, since live-cell phase contrast and fluorescence microscopy did not reveal clear differences between the morphology of nuclei of Saquinavir-treated and control cells, in contrast to cytoplasmic changes (Fig. 5D–G; Supplement Movie 12, Movie 13). Similar apoptotic changes in the nuclear ultrastructure were also observed in cryoXT data of Saquinar-treated HeLa cells transiently expressing GFP-tagged lamin B1 (Supplement Movie 14), and in a nucleus of an untreated cerebellar granule cell (not presented). Primary fibroblasts transiently expressing GFP-tagged lamin B1 and treated with Saquinavir were studied before by fluorescence and conventional transmission electron microscopy, but did not reveal apoptotic events [31], [32]. Thus, cryoXT, providing direct soft X-ray absorption contrast of natively preserved cellular components and compartments in their full three-dimensional expansion, might be even better suited to detect apoptosis in the nucleus than conventional transmission electron microscopy relying on heavy-metal adsorption of structures in thin sections, considered as gold standard in apoptosis research so far [68].

In this project, it was not our principal aim to study apoptosis/programmed cell death by cryoXT. Observing the described changes in the nuclear ultrastructure in different cell types might indicate a broader relevance of these data for research on this important cell biological process [69]. Thus, we decided to present this preliminary findings to document the potential of cryoXT for apoptosis research, perhaps in further studies employing defined model systems in an integrative approach of biochemical and imaging methods (see, for instance [70], [71]). In fact, other cryoXT studies on nuclear ultrastructure have shown already comparable features without connecting it to apoptotic events [72], [73]. Unfortunately, in these reports the spatial resolution does not allow to support that speculation, by discriminating, for instance, the two membranes of the nuclear envelope from each other and/or from boundary layers around condensed chromatin, as shown here (Fig. 5B and C; Supplement Movie 10, Movie 11). For investigating the nature of the observed boundary layers in apoptotic nuclei, also other X-ray cryo-imaging methods might be applied: X-ray fluorescence tomography or energy-specific X-ray scanning microscopy might help to elucidate by nanoscale elemental/chemical mapping if, for instance, phosphor is enriched in these structures (for methodological review, see Ref. [2]).

From a methodological point of view, data presented here emphasize the advantages of cryoXM as compared to fluorescence microscopy. In cryo-immobilized samples, cryoXM/T provides high-contrast three-dimensional structural data based on absorption contrast, at a resolution better than super resolution in fluorescence microscopy, not relying on fluorophore-tagged cell components. Thus, a complete cell can be imaged in its native structural state, making cryoXM/T together with other emerging cryo-imaging approaches like focused ion beam milling for serial block face scanning electron microscopy in vitreous samples [74] a useful technique in an integrative toolbox of imaging methods for cell biology research [75].

## 5 Conclusions

An elaborate three-dimensional network of the LBR1TM-GFP-labeled NR was imaged in nuclei of Saquinavir-treated rabbit kidney cells by correlative cryoFM/XT on flat sample grids. In these experiments, newly designed polyelectrolyte-Qdot^®^ 605 coated gold beads were successfully employed as alignment and correlation markers. In many of the cells treated with a moderate Saquinavir concentration, however, compacted and segregated chromatin masked other structural changes in the nucleus. These dramatic rearrangements in the nuclear ultrastructure were interpreted as caused by programmed cell death/apoptosis. The presented data recommend cryoXT as an auspicious imaging technique in apoptosis research, especially in the cell nucleus.

## Appendix A Supporting information

Movie 1 Prealigned cryoXT tilt series corresponding to data shown in Fig. 1A.

.

Movie 2 Prealigned cryoXT tilt series corresponding to data shown in Fig. 1C.

Movie 3 Sliced view through the tomographic reconstruction corresponding to data shown in Fig. 1D.

Movie 4 Inverse contrast volume-rendered tomographic reconstruction corresponding to data shown in Fig. 1E

Movie 5 Dynamic superimposition of in-column fluorescence (Fig. 3D) and cryoXT data (Fig. 3E).

Movie 6 Sliced view through the tomographic reconstruction corresponding to data shown in Fig. 3E.

Movie 7 Prealigned cryoXT tilt series corresponding to data shown in Fig. 4A.

Movie 8 Dynamic superimposition of in-column fluorescence cryo-images taken before (Fig. 4B) and after cryoXT tilt series acquisition (Fig. 4C).

Movie 9 Dynamic superimposition of in-column fluorescence cryo-images taken before (Fig. 4D) and after cryoXT tilt series acquisition (Fig. 4E).

Movie 10 Sliced view through the tomographic reconstruction corresponding to data shown in Fig. 5B.

Movie 11 Sliced view through the tomographic reconstruction corresponding to data shown in Fig. 5C.

Movie 12 Dynamic superimposition of live-cell microscopic phase contrast (Fig. 5D) and fluorescence images of a control sample (Fig. 5E).

Movie 13 Dynamic superimposition of live-cell microscopic phase contrast (Fig. 5F) and fluorescence images of a Saquinavir-treated sample (Fig. 5G).

Movie 14 Sliced view through the tomographic reconstruction of the vitreous nucleus of a HeLa cell transiently transfected to express GFP-lamin B1 and incubated with 20 µM Saquinavir for 48 h.

## Footnotes

Appendix A Supplementary data associated with this article can be found in the online version at doi:10.1016/j.ultramic.2014.05.009.

## References

[1] Gierasch L.M., Gershenson A. (2009). Post-reductionist protein science, or putting Humpty Dumpty back together again. Nat. Chem. Biol..

[2] Falcone R., Jacobsen C., Kirz J., Marchesini S., Shapiro D., Spence J. (2011). New directions in X-ray microscopy. Contemp. Phys..

[3] Schneider G., Guttmann P., Rehbein S., Werner S., Follath R. (2012). Cryo X-ray microscope with flat sample geometry for correlative fluorescence and nanoscale tomographic imaging. J. Struct. Biol..

[4] Hertz H.M., von Hofsten O., Bertilson M., Vogt U., Holmberg A., Reinspach J., Martz D., Selin M., Christakou A.E., Jerlström-Hultqvist J., Svard S. (2012). Laboratory cryo soft X-ray microscopy. J. Struct. Biol..

[5] Martz D.H., Selin M., von Hofsten O., Fogelqvist E., Holmberg A., Vogt U., Legall H., Blobel G., Seim C., Stiel H., Hertz H.M. (2012). High average brightness water window source for short-exposure cryomicroscopy. Opt. Lett..

[6] Guttmann P., Zeng X., Feser M., Heim S., Yun W., Schneider G., David C., Nolting F., Quitmann C., Stampanoni M., Pfeiffer F. (2009). Proceedings of the 9th International Conference on X-ray Microscopy.

[7] Chao W., Fischer P., Tyliszczak T., Rekawa S., Anderson E., Naulleau P. (2012). Real space soft X-ray imaging at 10 nm spatial resolution. Opt. Express.

[8] Rehbein S., Guttmann P., Werner S., Schneider G. (2012). Characterization of the resolving power and contrast transfer function of a transmission X-ray microscope with partially coherent illumination. Opt. Express.

[9] Parkinson D.Y., Epperly L.R., McDermott G., Le Gros M.A., Boudreau R.M., Larabell C.A., Sousa A.A., Kruhlak M.J. (2013).

[10] McDermott G., Le Gros M.A., Larabell C.A. (2012). Visualizing cell architecture and molecular location using soft X-ray tomography and correlated cryo-light microscopy. Annu. Rev. Phys. Chem..

[11] Lučić V., Rigort A., Baumeister W. (2013). Cryo-electron tomography: the challenge of doing structural biology *in situ*. J. Cell Biol..

[12] Saxton W.O., Baumeister W., Hahn M. (1984). Three-dimensional reconstruction of imperfect two-dimensional crystals. Ultramicroscopy.

[13] Nickell S., Hegerl R., Baumeister W., Rachel R. (2003). *Pyrodictium cannulae* enter the periplasmic space but do not enter the cytoplasm, as revealed by cryo-electron tomography. J. Struct. Biol..

[14] Chen Y., Förster F. (2014). Iterative reconstruction of cryo-electron tomograms using nonuniform fast Fourier transforms. J. Struct. Biol..

[15] Guesdon A., Blestel S., Kervrann C., Chretien D. (2013). Single *versus* dual-axis cryo-electron tomography of microtubules assembled *in vitro*: limits and perspectives. J. Struct. Biol..

[16] Mastronarde D.N. (1997). Dual-axis tomography: an approach with alignment methods that preserve resolution. J. Struct. Biol..

[17] Weiss D., Schneider G., Niemann B., Guttmann P., Rudolph D., Schmahl G. (2000). Computed tomography of cryogenic biological specimens based on X-ray microscopic images. Ultramicroscopy.

[18] Uchida M., Sun Y., McDermott G., Knoechel C., Le Gros M.A., Parkinson D., Drubin D.G., Larabell C.A. (2011). Quantitative analysis of yeast internal architecture using soft X-ray tomography. Yeast.

[19] Hanssen E., Knoechel C., Dearnley M., Dixon M.W.A., Le Gros M., Larabell C., Tilley L. (2012). Soft X-ray microscopy analysis of cell volume and hemoglobin content in erythrocytes infected with asexual and sexual stages of *Plasmodium falciparum*. J. Struct. Biol..

[20] Garvalov B.K., Zuber B., Bouchet-Marquis C., Kudryashev M., Gruska M., Beck M., Leis A., Frischknecht F., Bradke F., Baumeister W., Dubochet J., Cyrklaff M. (2006). Luminal particles within cellular microtubules. J. Cell Biol..

[21] Lučić V., Kossel A.H., Yang T., Bonhoeffer T., Baumeister W., Sartori A. (2007). Multiscale imaging of neurons grown in culture: from light microscopy to cryo-electron tomography. J. Struct. Biol..

[22] Ibiricu I., Huiskonen J.T., Döhner K., Bradke F., Sodeik B., Grünewald K. (2011). Cryo electron tomography of herpes simplex virus during axonal transport and secondary envelopment in primary neurons. PLoS Pathog..

[23] Chichón F.J., Rodríguez M.J., Pereiro E., Chiappi M., Perdiguero B., Guttmann P., Werner S., Rehbein S., Schneider G., Esteban M., Carrascosa J.L. (2012). Cryo X-ray nano-tomography of vaccinia virus infected cells. J. Struct. Biol..

[24] Rigort A., Bäuerlein F.J.B., Villa E., Eibauer M., Laugks T., Baumeister W., Plitzko J.M. (2012). Focused ion beam micromachining of eukaryotic cells for cryoelectron tomography. Proc. Natl. Acad. Sci. U. S. A..

[25] Hsieh C., Schmelzer T., Kishchenko G., Wagenknecht T., Marko M. (2014). Practical workflow for cryo focused-ion-beam milling of tissues and cells for cryo-TEM tomography. J. Struct. Biol..

[26] McDermott G., Fox D.M., Epperly L., Wetzler M., Barron A.E., Le Gros M.A., Larabell C.A. (2012). Visualizing and quantifying cell phenotype using soft X-ray tomography. Bioessays.

[27] Cinquin B.P., Do M., McDermott G., Walters A.D., Myllys M., Smith E.A., Cohen-Fix O., Le Gros M.A., Larabell C.A. (2014). Putting molecules in their place. J. Cell. Biochem..

[28] Klupp B.G., Granzow H., Fuchs W., Keil G.M., Finke S., Mettenleiter T.C. (2007). Vesicle formation from the nuclear membrane is induced by coexpression of two conserved herpesvirus proteins. Proc. Natl. Acad. Sci. U. S. A..

[29] Hagen C., Guttmann P., Klupp B., Werner S., Rehbein S., Mettenleiter T.C., Schneider G., Grünewald K. (2012). Correlative VIS-fluorescence and soft X-ray cryo-microscopy/tomography of adherent cells. J. Struct. Biol..

[30] Mettenleiter T.C., Müller F., Granzow H., Klupp B.G. (2013). The way out: what we know and do not know about herpesvirus nuclear egress. Cell Microbiol..

[31] Goulbourne C.N., Vaux D.J. (2010). HIV protease inhibitors inhibit FACE1/ZMPSTE24: a mechanism for acquired lipodystrophy in patients on highly active antiretroviral therapy?. Biochem. Soc. Trans..

[32] Malhas A., Goulbourne C., Vaux D.J. (2011). The nucleoplasmic reticulum: form and function. Trends Cell Biol..

[33] Goulbourne C.N., Malhas A.N., Vaux D.J. (2011). The induction of a nucleoplasmic reticulum by prelamin A accumulation requires CTP:phosphocholine cytidylyltransferase-alpha. J. Cell Sci..

[34] Carregal-Romero S., Caballero-Díaz E., Beqa L., Abdelmonem A.M., Ochs M., Hühn D., Suau B.S., Valcarcel M., Parak W.J., Cooks R.G., Pemberton J.E. (2013).

[35] Wang S.J., Zhou C.H., Yuan H., Shen H.B., Zhao W.X., Ma L., Li L.S. (2013). A robust ligand exchange approach for preparing hydrophilic, biocompatible photoluminescent quantum dots. Mater. Res. Bull..

[36] Ellenberg J., Siggia E.D., Moreira J.E., Smith C.L., Presley J.F., Worman H.J., Lippincott-Schwartz J. (1997). Nuclear membrane dynamics and reassembly in living cells: targeting of an inner nuclear membrane protein in interphase and mitosis. J. Cell Biol..

[37] Graham F.L., van der Eb A.J. (1973). New technique for assay of infectivity of human adenovirus 5 DNA. Virology.

[38] Maske C.P., Hollinshead M.S., Higbee N.C., Bergo M.O., Young S.G., Vaux D.J. (2003). A carboxyl-terminal interaction of lamin B1 is dependent on the CAAX endoprotease Rce1 and carboxymethylation. J. Cell Biol..

[39] McLean K., VanDeVen N.A., Sorenson D.R., Daudi S., Liu R. (2009). The HIV protease inhibitor saquinavir induces endoplasmic reticulum stress, autophagy, and apoptosis in ovarian cancer cells. Gynecol. Oncol..

[40] Zhou C.H., Yuan H., Shen H.B., Guo Y., Li X.M., Liu D., Xu L., Ma L., Li L.S. (2011). Synthesis of size-tunable photoluminescent aqueous CdSe/ZnS microspheres *via* a phase transfer method with amphiphilic oligomer and their application for detection of HCG antigen. J. Mater. Chem..

[41] Yu X., Lei D.Y., Amin F., Hartmann R., Acuna G.P., Guerrero-Martínez A., Maier S.A., Tinnefeld P., Carregal-Romero S., Parak W.J. (2013). Distance control in-between plasmonic nanoparticles *via* biological and polymeric spacers. Nano Today.

[42] Caruso F., Niikura K., Furlong D.N., Okahata Y. (1997). 1. Ultrathin multilayer polyelectrolyte films on gold: construction and thickness determination. Langmuir.

[43] Gueroui Z., Libchaber A. (2004). Single-molecule measurements of gold-quenched quantum dots. Phys. Rev. Lett..

[44] Ma X.D., Fletcher K., Kipp T., Grzelczak M.P., Wang Z., Guerrero-Martínez A., Pastoriza-Santos I., Kornowski A., Liz-Marzán L.M., Mews A. (2011). Photoluminescence of individual Au/CdSe nanocrystal complexes with variable interparticle distances. J. Phys. Chem. Lett..

[45] Donath E., Sukhorukov G.B., Caruso F., Davis S.A., Mohwald H. (1998). Novel hollow polymer shells by colloid-templated assembly of polyelectrolytes. Angew. Chem. Int. Ed..

[46] Schneider G., Decher G., Nerambourg N., Praho R., Werts M.H.V., Blanchard-Desce M. (2006). Distance-dependent fluorescence quenching on gold nanoparticles ensheathed with layer-by-layer assembled polyelectrolytes. Nano Lett..

[47] Amin F., Yushchenko D.A., Montenegro J.M., Parak W.J. (2012). Integration of organic fluorophores in the surface of polymer-coated colloidal nanoparticles for sensing the local polarity of the environment. ChemPhysChem.

[48] Schneider G., Guttmann P., Heim S., Rehbein S., Mueller F., Nagashima K., Heymann J.B., Müller W.G., McNally J.G. (2010). Three-dimensional cellular ultrastructure resolved by X-ray microscopy. Nat. Methods.

[49] Rigort A., Bäuerlein F.J.B., Leis A., Gruska M., Hoffmann C., Laugks T., Böhm U., Eibauer M., Gnaegi H., Baumeister W., Plitzko J.M. (2010). Micromachining tools and correlative approaches for cellular cryo-electron tomography. J. Struct. Biol..

[50] Kremer J.R., Mastronarde D.N., McIntosh J.R. (1996). Computer visualization of three-dimensional image data using IMOD. J. Struct. Biol..

[51] Mastronarde D.N. (2008). Correction for non-perpendicularity of beam and tilt axis in tomographic reconstructions with the IMOD package. J. Microsc..

[52] Graf C., Meinke M., Gao Q., Hadam S., Raabe J., Sterry W., Blume-Peytavi U., Lademann J., Ruhl E., Vogt A. (2009). Qualitative detection of single submicron and nanoparticles in human skin by scanning transmission X-ray microscopy. J. Biomed. Opt..

[53] Sorzano C.O.S., Messaoudi C., Eibauer M., Bilbao-Castro J.R., Hegerl R., Nickell S., Marco S., Carazo J.M. (2009). Marker-free image registration of electron tomography tilt-series. BMC Bioinform..

[54] Hummel E., Guttmann P., Werner S., Tarek B., Schneider G., Kunz M., Frangakis A.S., Westermann B. (2012). 3d ultrastructural organization of whole *Chlamydomonas reinhardtii* cells studied by nanoscale soft X-ray tomography. PLoS One.

[55] Parkinson D.Y., Knoechel C., Yang C., Larabell C.A., Le Gros M.A. (2012). Automatic alignment and reconstruction of images for soft X-ray tomography. J. Struct. Biol..

[56] Schorb M., Briggs J.A.G. (2014). Correlated cryo-fluorescence and cryo-electron microscopy with high spatial precision and improved sensitivity. Ultramicroscopy.

[57] Schellenberger P., Kaufmann R., Siebert C.A., Hagen C., Wodrich H., Grünewald K. (2014). High-precision correlative fluorescence and electron cryo microscopy using two independent alignment markers. Ultramicroscopy.

[58] Ma X.D., Tan H., Kipp T., Mews A. (2010). Fluorescence enhancement, blinking suppression, and gray states of individual semiconductor nanocrystals close to gold nanoparticles. Nano Lett..

[59] Steinbrener J.F. (2006).

[60] Moser S.K. (2008).

[61] Böhm U. (2010).

[62] Duke E.M.H., Razi M., Weston A., Guttmann P., Werner S., Henzler K., Schneider G., Tooze S.A., Collinson L.M. (2014). Imaging endosomes and autophagosomes in whole mammalian cells using correlative cryo-fluorescence and cryo-soft X-ray microscopy (cryo-CLXM). Ultramicroscopy.

[63] Briegel A., Chen S.Y., Koster A.J., Plitzko J.M., Schwartz C.L., Jensen G.J., Jensen G. (2010).

[64] Le Gros M.A., McDermott G., Uchida M., Knoechel C.G., Larabell C.A. (2009). High-aperture cryogenic light microscopy. J. Microsc..

[65] Pajonk F., Himmelsbach J., Riess K., Sommer A., McBride W.H. (2002). The human immunodeficiency virus (HIV)-1 protease inhibitor Saquinavir inhibits proteasome function and causes apoptosis and radiosensitization in non-HIV-associated human cancer cells. Cancer Res..

[66] Elmore S. (2007). Apoptosis: a review of programmed cell death. Toxicol. Pathol..

[67] Dominguez F., Cejudo F.J. (2012). A comparison between nuclear dismantling during plant and animal programmed cell death. Plant Sci..

[68] Ulukaya E., Acilan C., Ari F., Ikitimur E., Yilmaz Y. (2011). A glance at the methods for detection of apoptosis qualitatively and quantitatively. Turk. J. Biochem..

[69] Fuchs Y., Steller H. (2011). Programmed cell death in animal development and disease. Cell.

[70] Toné S., Sugimoto K., Tanda K., Suda T., Uehira K., Kanouchi H., Samejima K., Minatogawa Y., Earnshaw W.C. (2007). Three distinct stages of apoptotic nuclear condensation revealed by time-lapse imaging, biochemical and electron microscopy analysis of cell-free apoptosis. Exp. Cell Res..

[71] Karreman M.A., Agronskaia A.V., Verkleij A.J., Cremers F.F.M., Gerritsen H.C., Humbel B.M. (2009). Discovery of a new RNA-containing nuclear structure in UVC-induced apoptotic cells by integrated laser electron microscopy. Biol. Cell.

[72] Clowney E.J., LeGros M.A., Mosley C.P., Clowney F.G., Markenskoff-Papadimitriou E.C., Myllys M., Barnea G., Larabell C.A., Lomvardas S. (2012). Nuclear aggregation of olfactory receptor genes governs their monogenic expression. Cell.

[73] Isaacson S.A., Larabell C.A., Le Gros M.A., McQueen D.M., Peskin C.S. (2013). The influence of spatial variation in chromatin density determined by X-ray tomograms on the time to find DNA binding sites. Bull. Math. Biol..

[74] Schertel A., Snaidero N., Han H.M., Ruhwedel T., Laue M., Grabenbauer M., Möbius W. (2013). Cryo FIB-SEM: volume imaging of cellular ultrastructure in native frozen specimens. J. Struct. Biol..

[75] Zeev-Ben-Mordehai T., Hagen C., Grünewald K. (2014). A cool hybrid approach to the herpesvirus ‘life’ cycle. Curr. Opin. Virol..

