# Supplementary figures and images for: Multimodal nanoparticles as alignment and correlation markers in fluorescence/soft X-ray cryo-microscopy/tomography of nucleoplasmic reticulum and apoptosis in mammalian cells
Source: Ultramicroscopy. 2014 Nov;146:46–54. doi: 10.1016/j.ultramic.2014.05.009 (PMC4181793; doi:10.1016/j.ultramic.2014.05.009)

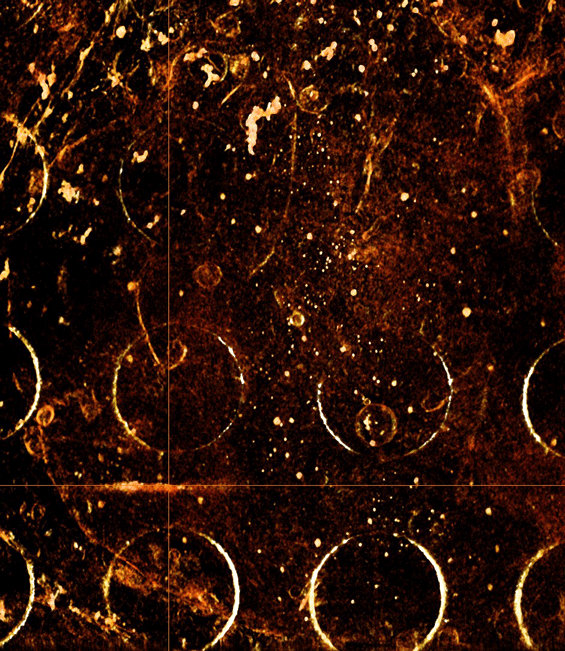

Supplement: Movie 5 — Dynamic superimposition of in-column fluorescence (Fig. 3D) and cryoXT data (Fig. 3E). [file mmc5.gif]

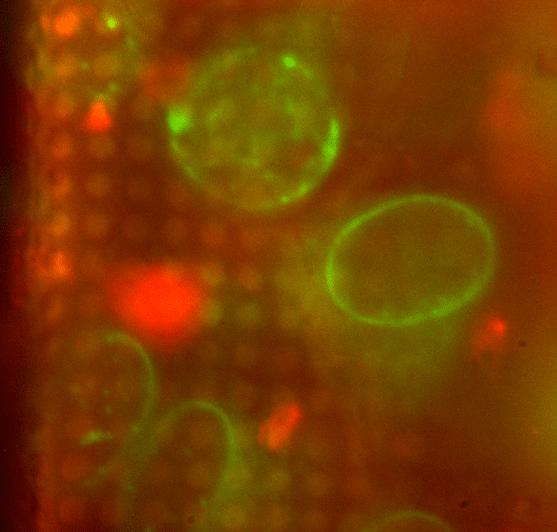

Supplement: Movie 8 — Dynamic superimposition of in-column fluorescence cryo-images taken before (Fig. 4B) and after cryoXT tilt series acquisition (Fig. 4C). [file mmc8.gif]

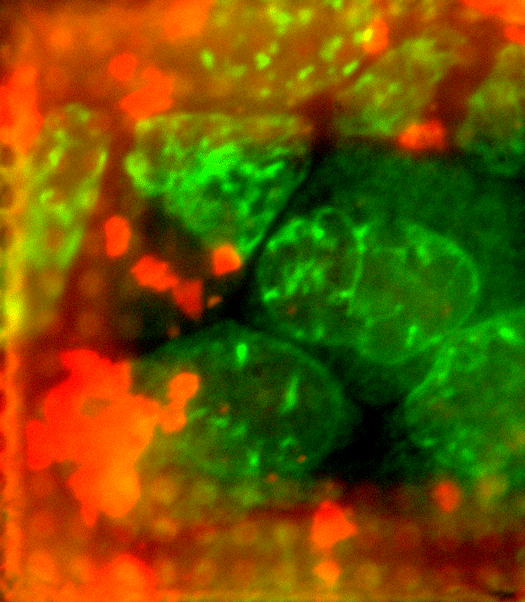

Supplement: Movie 9 — Dynamic superimposition of in-column fluorescence cryo-images taken before (Fig. 4D) and after cryoXT tilt series acquisition (Fig. 4E). [file mmc9.gif]

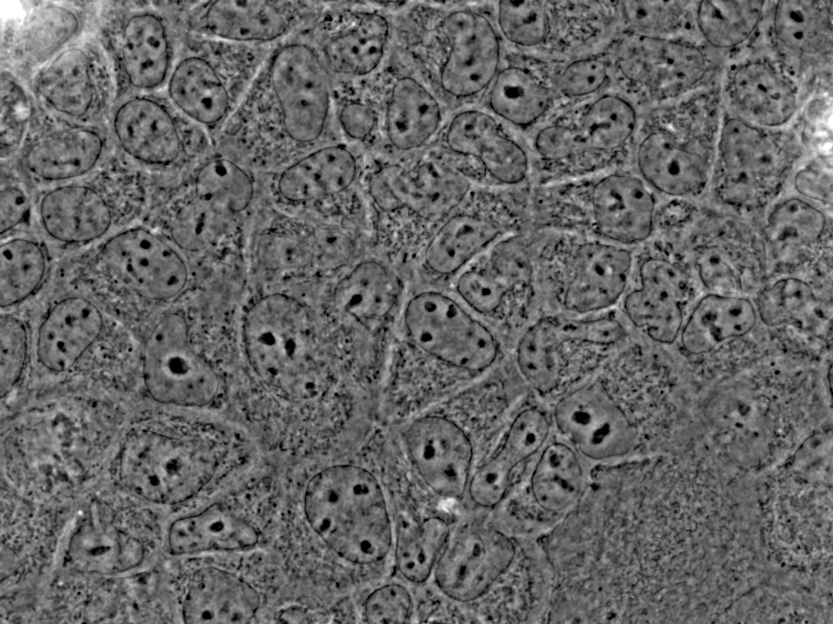

Supplement: Movie 12 — Dynamic superimposition of live-cell microscopic phase contrast (Fig. 5D) and fluorescence images of a control sample (Fig. 5E). [file mmc12.gif]

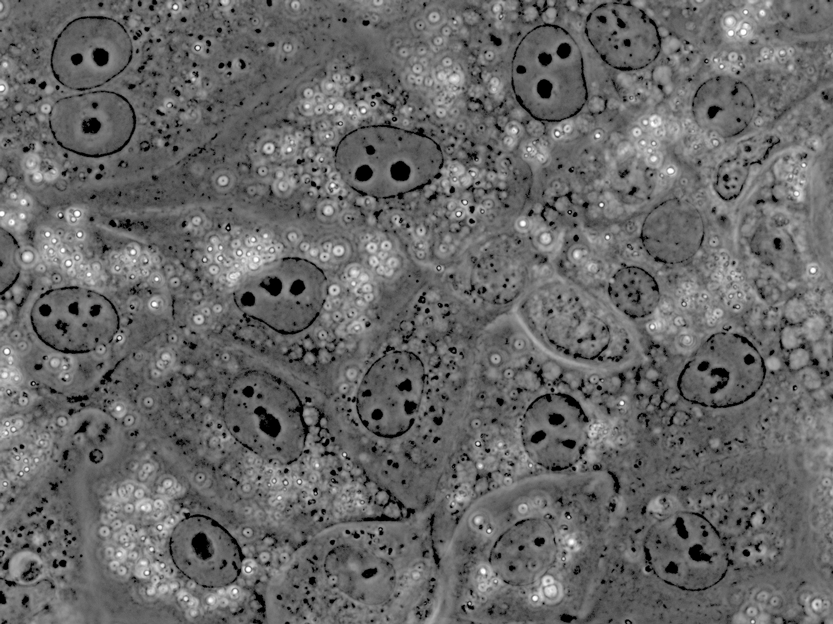

Supplement: Movie 13 — Dynamic superimposition of live-cell microscopic phase contrast (Fig. 5F) and fluorescence images of a Saquinavir-treated sample (Fig. 5G). [file mmc13.gif]
