# Supplementary material for: Multimodal nanoparticles as alignment and correlation markers in fluorescence/soft X-ray cryo-microscopy/tomography of nucleoplasmic reticulum and apoptosis in mammalian cells
Source: Ultramicroscopy. 2014 Nov;146:46–54. doi: 10.1016/j.ultramic.2014.05.009 (PMC4181793; doi:10.1016/j.ultramic.2014.05.009)
Supplement: Supplementary file 15 — Supplementary data [file mmc15.docx]

**Multimodal nanoparticles as alignment and correlation markers in fluorescence/soft X-ray cryo-microscopy/tomography of nucleoplasmic reticulum and apoptosis in mammalian cells**

Christoph Hagen, Stephan Werner, Susana Carregal-Romero, Ashraf N. Malhas, Barbara Klupp, Peter Guttmann, Stefan Rehbein, Katja Henzler, Thomas C. Mettenleiter, David J. Vaux, Wolfgang J. Parak, Gerd Schneider, Kay Grünewald

SUPPORTING INFORMATION

1. Protocol for the synthesis and characterization of polyelectrolyte- Qdot^®^ 605 coated gold beads
2. Protocol for the synthesis and characterization of polyelectrolyte-Qdot^®^ 605 coated gold beads

Commercial gold beads (Cytodiagnostics # CG-250-10) were wrapped (before being coated with quantum dots) with nine different layers of polyelectrolytes with alternating charge *via* the Layer-by-Layer (LbL) approach as it follows: 10 mL of the commercial solution of gold beads with a concentration of 7.1x10^8^ particles/mL were washed thrice with Milli-Q water. After the last centrifugation step, particles were re-dispersed in 10 mL of a 0.2 mg/mL solution of poly(allylamine hydrochloride) (PAH) (M_w_ ~15 kDa, Aldrich # 283215) at pH 6.5 and shaken during 10 min. After that, Au beads were washed twice with Milli-Q water and then coated with poly(styrene sulfonate) (PSS) (M_w_ ~14.9 kDa, Polymer Standards Service # pss18061-2). The coating with the negative polyelectrolyte PSS was performed also with 10 mL of a solution 0.2 mg/mL of PSS at pH 6.5 during 10 min. During the washing steps the sample was precipitated by gently centrifugation (1,500 rpm for 20 min). The LbL coating was repeated until the gold beads had nine layers of polyelectrolytes (Au@(PAH/PSS)_4_ PAH). After that, the negatively charged quantum dots (Qdot^®^ 605) were attached to the coated gold beads *via* electrostatic interactions. These red fluorescent nanoparticles (*i.e*. Qdot^®^ 605 nanoparticles) were commercial (Invitrogen # Q21701MP) and initially hydrophobic. Thus a polymer coating procedure was performed, as it has been previously reported, to render the particles soluble in water and negatively charged [1]. 100 µL of a water solution of quantum dots (1.3 µM) were mixed with the gold beads dissolved in 1 mL of Milli-Q water and shaken during 30 min. After this time, the sample was again washed twice with water, and the last layer of PSS was added. Finally the sample was washed and re-dispersed in 2 mL of Milli-Q water. The sample of multilayer polyelectrolyte-Qdot^®^ 605 coated beads was characterized with transmission electron microscopy (TEM), dynamic light scattering (DLS), fluorescence spectroscopy and absorption spectrophotometry.

The assembly of polyelectrolyte layers on the gold beads was confirmed by measuring the change of ζ-potential after the assembly of the first six layers (Figure SI.1.1A). The final value of ζ-potential for the Au@(PAH/PSS)_4_ PAH Qdot^®^ 605 PSS beads was -9.5 ± 0.9 mV. Absorption spectra during the LbL process were also taken. Figure SI.1.1B shows the absorption spectra of the Au beads before coating (black line), after the assembly of four layers of polyelectrolytes (red line), after the assembly of Qdot^®^ 605 nanoparticles (blue line) and after the assembly of the last layer of polyelectrolyte (PSS; green line). As it can be seen, the presence of polyelectrolyte layers on the surface of gold beads slightly modified the absorption spectra, and the attachment of quantum dots increased the absorption of the core-shell structure at low wavelengths between 250 and 500 nm.

**Figure SI.1.1.** (A) Change of ζ-potential after the assembly of *n* number of polyelectrolyte layers during the production of polyelectrolyte-Qdot^®^ 605 coated gold beads. (B) Absorption spectra of Au beads (black line), Au@(PAH/PSS)_2_ (red line), Au@(PAH/PSS)_4_ PAH Qdot^®^ 605 (blue line) and Au@(PAH/PSS)_4_ PAH Qdot^®^ 605 PSS (green line).

In Figure SI.1.2, transmission electron microscopy (TEM) images of the gold beads and the gold beads after the assembly of ten layers of polyelectrolytes and quantum dots are shown. The presence of the polyelectrolytes and quantum dots was not evident in the images but the increase of the hydrodynamic diameter measured in the DLS (Figure SI.1.2D) from 210 ± 2 nm to 277 ± 4 nm indicated the presence the multilayer film.


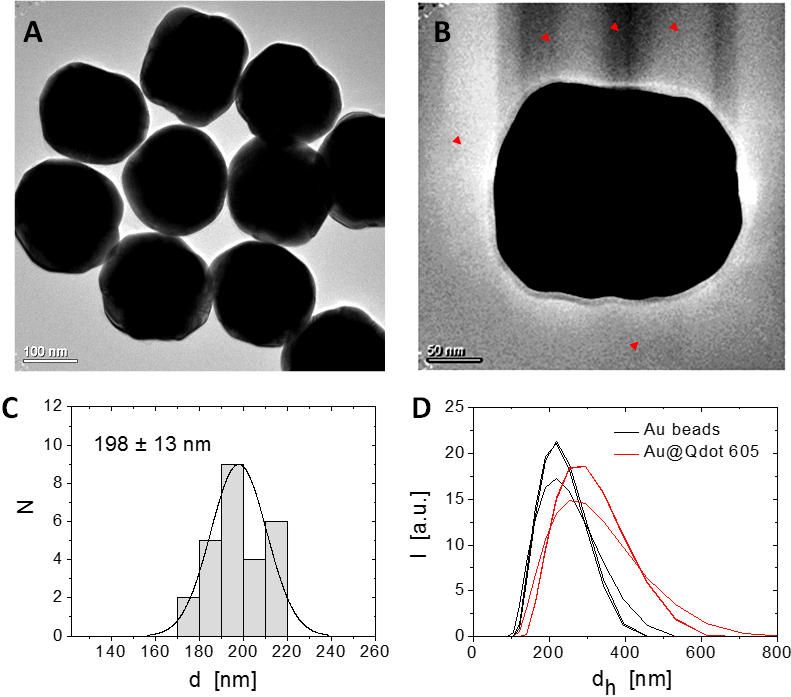

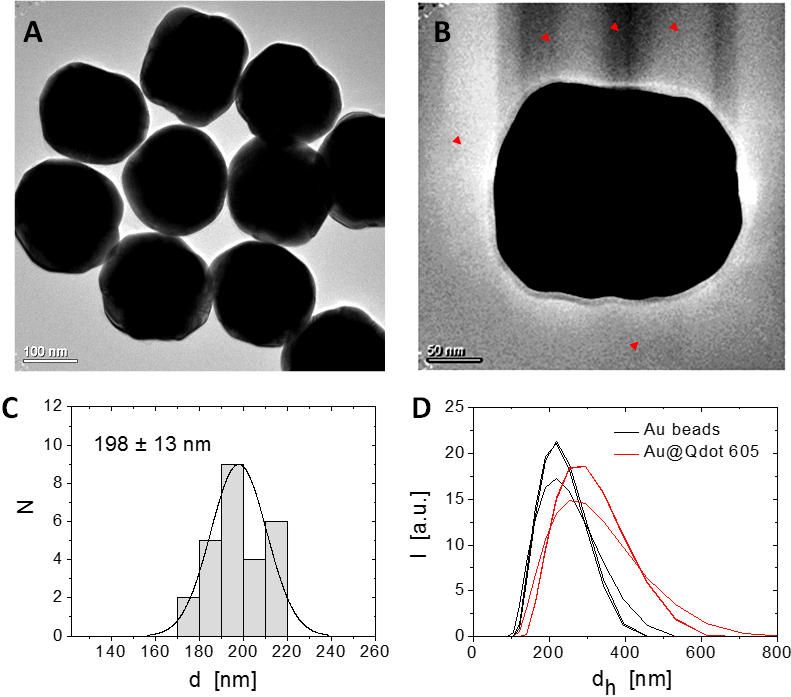

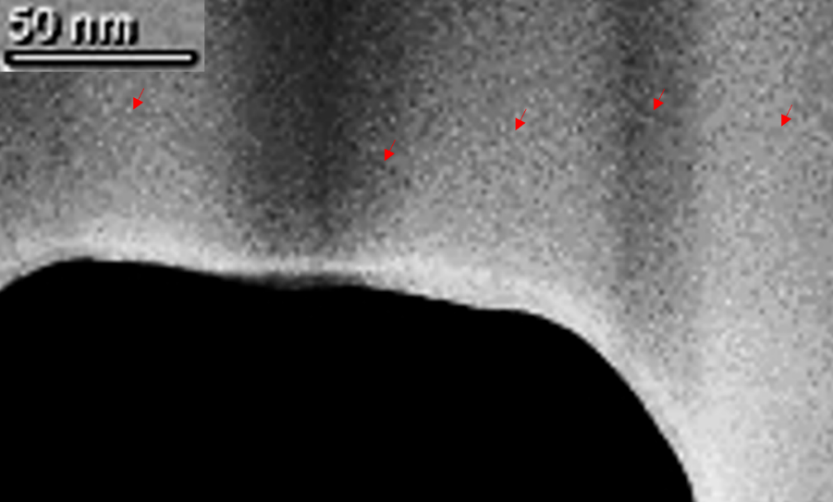


**B**

**Figure SI.1.2.** (A) TEM image of the commercial gold beads. The scale bar corresponds to 100 nm. (B) TEM image of polyelectrolyte-Qdot^®^ 605 coated gold bead. The red arrows mark faint structures that might present the edge of the polyelectrolyte shell. The scale bar corresponds to 50 nm. (C) Size distribution of the gold beads (inorganic core diameter) as obtained from TEM images. (D) Hydrodynamic diameter of gold beads before (black line) and after assembly of polyelectrolytes and quantum dots (red line) measured thrice in Milli-Q water, as determined by dynamic light scattering (DLS)

In Figure SI.1.3A we show images of the final 2 mL solution of polyelectrolyte-Qdot^®^ 605 coated gold beads illuminated with a UV lamp where the red fluorescence is evident, and an image of the particles after sedimentation. The absorption spectra compared with the fluorescence spectra is shown in Figure SI.1.3B.

**Figure SI.1.3.** (A) Pictures of the polyelectrolyte-Qdot^®^ 605 coated gold beads under illumination with a UV lamp (left side) and with natural light (right side) after sedimentation. (B) Absorption and fluorescence spectra of the prepared polyelectrolyte-Qdot^®^ 605 coated gold beads.

[1] F. Amin, D.A. Yushchenko, J.M. Montenegro, W.J. Parak, Integration of organic fluorophores in the surface of polymer-coated colloidal nanoparticles for sensing the local polarity of the environment, ChemPhysChem 13 (2012) 1030-1035.
